# Teplizumab’s immunomodulatory effects on pancreatic β-cell function in type 1 diabetes mellitus

**DOI:** 10.1186/s40842-024-00181-w

**Published:** 2024-08-10

**Authors:** Emmanuel Kokori, Gbolahan Olatunji, Ikponmwosa Jude Ogieuhi, John Ehi Aboje, Doyin Olatunji, Sikiru Ademola Aremu, Stephen Chukwuemeka Igwe, Abdulrahmon Moradeyo, Yusuf Ismaila Ajayi, Nicholas Aderinto

**Affiliations:** 1 Department of Medicine and Surgery, University of Ilorin, Ilorin, Nigeria; 2 Siberian State Medical University, Tomsk, Russia; 3 Department of Medicine, College of Health Sciences, Benue State University, Benue, Nigeria; 4 Department of Health Sciences, Western Illinois University, Macomb, USA; 5 Department of Medicine and Surgery, Ladoke Akintola University of Technology, Ogbomoso, Nigeria; 6 Department of Medicine and Surgery, Obafemi Awolowo University, Ife, Nigeria

**Keywords:** Teplizumab, Type 1 diabetes memploys a narrative approachellitus, Pancreatic β-cells, Immunomodulation, CD3 antigen

## Abstract

This review explores the immunomodulatory potential of Teplizumab and its impact on pancreatic β-cell function in T1D. Characterized by the autoimmune destruction of insulin-producing beta cells, T1D’s management involves maintaining glycemic control through exogenous insulin. Teplizumab, a humanized monoclonal antibody targeting the CD3 antigen, has shown promise in delaying T1D onset and preserving residual β-cell function. The review employs a narrative approach, synthesizing evidence from diverse clinical trials and studies gathered through a meticulous literature search. It scrutinizes Teplizumab’s mechanisms of action, including its influence on autoreactive CD8 + T cells and regulatory T cells, offering insights into its immunological pathways. The synthesis of findings from various trials demonstrates Teplizumab’s efficacy in preserving C-peptide levels and reducing exogenous insulin requirements, particularly in recent-onset T1D. Considering Teplizumab’s real-world implications, the paper addresses potential obstacles, including side effects, patient selection criteria, and logistical challenges. It also emphasizes exploring combination therapies and personalized treatment strategies to maximize Teplizumab’s benefits. The review contributes a nuanced perspective on Teplizumab’s clinical implications and future directions in T1D management, bridging theoretical understanding with practical considerations.

## Introduction

Type 1 diabetes mellitus (T1D) is a complex autoimmune disorder characterized by the progressive destruction of insulin-producing pancreatic beta cells [1]. This condition exhibits heterogeneity in metabolic, genetic, and immunogenetic aspects, necessitating personalized approaches, particularly due to age-related differences [2]. The loss of insulin secretion in T1D can occur rapidly or gradually, with adults often retaining residual insulin production (detectable/higher c-peptide), leading to improved glycemic control. In contrast, youth with T1D are more susceptible to diabetic ketoacidosis [1, 2]. Heterogeneity is also evident in the presence of other autoimmune conditions, obesity, comorbidities, and the development of diabetes-related complications [3, 4]. Within Type 1 diabetes (T1D), beta cells within pancreatic islets undergo autoimmune destruction over an extended period, resulting in a complete lack of insulin [3]. While the precise cause remains elusive, a genetic predisposition is suspected, particularly linked to specific HLA (DR and DQ) alleles, with a more significant association in youth-onset T1D. Various genes contribute to heritability [1, 5, 6].

Potential triggers for autoimmune beta-cell destruction in at-risk individuals include viruses, environmental factors, dietary elements, and other stressors [7–9]. Studies have indicated an elevated T1D risk associated with infections such as Coxsackie virus, enteroviruses, cytomegalovirus, rubella virus, influenza B, mumps virus, and more recently, SARS-CoV-2 (COVID-19) [1, 7–9]. The presence of circulating pancreatic islet autoantibodies, including islet cell cytoplasmic antibodies (ICA), insulin antibodies (IAAs), glutamic acid decarboxylase isoform 65 (GAD65), insulinoma antigen 2/islet tyrosine phosphatase 2 (IA-2), and zinc transporter isoform 8 (ZnT8), indicates T1D risk or development [10]. IAAs are predominantly found in children [1, 11], while GAD65 is the most common autoantibody in adults [4]. The number and titers of detectable antibodies correlate with the risk of T1D development. Effectively managing T1D involves multiple daily insulin injections (MDI), insulin pump therapy, automated insulin delivery systems, and glucose monitoring, ideally through continuous glucose monitors (CGM) [12–15].

Teplizumab, a humanized monoclonal antibody (mAb), received FDA approval on November 17, 2022, as the inaugural immunoprevention agent sanctioned to delay the onset of T1DM in individuals aged 8 and above [16]. This delay offers advantages, including decreased insulin dependency, fewer hospital visits, reduced complications, improved glucose control, and a prolonged window for potential interventions [16]. It targets the differentiation 3 (CD3) antigen cluster, coexpressed with the T-cell receptor (TCR) on the surface of T lymphocytes. CD3 functions as a T-cell co-receptor, activating and differentiating CD8 and CD4 naive T-cells. Monoclonal antibodies targeting CD3, known as anti-CD3 antibodies, obstruct the interaction between CD3 and the T-cell receptor, preventing the activation of T cells, particularly CD8 + T lymphocytes crucial in beta-cell destruction [13]. Additionally, these antibodies elevate CD4 + Tregs, fostering self-tolerance [14]. Extensive research has delved into the anti-CD3 immunological pathway in Type 1 diabetes (T1D) [15]. Teplizumab is associated with a temporary systemic increase in the proportion of CD4 + Foxp3 + T cells, helping restore the balance between tolerogenic Tregs and pathogenic T-cells. Ongoing research explores the application of teplizumab in treating recent-onset T1DM [16].

This review is motivated by the need to comprehend the interplay between Teplizumab and pancreatic β-cell function in individuals with T1DM. Despite advancements, there remains a noticeable gap in the literature concerning an analysis of Teplizumab’s effects on pancreatic β-cells. Thus, this review aims to explore Teplizumab’s impact, critically evaluating its mechanisms of action, potential influence on the immune system, and practical applications. By addressing this gap, the review seeks to contribute significantly to the existing body of knowledge, offering insights for validating new-onset treatments and suggesting avenues for future refinements in diabetes management.

## Methodology

### Study design

This study employs a narrative approach to investigate the effects of Teplizumab on pancreatic β-cells in patients with T1DM. The methodology incorporates a thorough review of relevant literature, spanning primary research articles and clinical trials.

### Literature search strategy

A literature search is conducted across electronic databases, including but not limited to PubMed, Scopus, and Web of Science. Keywords such as “Teplizumab,” “Type 1 Diabetes Mellitus,” “pancreatic β-cells,” and related terms are employed to identify relevant articles. Boolean operators (AND, OR) and filters are utilized to refine the search results, ensuring the inclusion of studies focused on Teplizumab’s impact on pancreatic β-cell function in T1DM patients.

### Inclusion and exclusion criteria

The inclusion criteria for articles include studies published in peer-reviewed journals, clinical trials, and observational studies investigating Teplizumab’s effects on pancreatic β-cells. Articles are included if they provide insights into Teplizumab’s immunomodulatory mechanisms, clinical outcomes, and practical applications in the context of T1DM. Exclusion criteria involve non-English language publications, review articles without original data, and studies lacking relevance to Teplizumab’s impact on pancreatic β-cells. No time limit was used in the literature search.

### Data extraction

Data extraction is conducted systematically from selected articles. Key information includes study design, participant characteristics, Teplizumab dosage and administration, outcomes related to pancreatic β-cell function, and any reported adverse effects.

### Data synthesis and analysis

Data synthesis involves a qualitative summary of study findings. A narrative synthesis is employed to integrate and interpret the results. Themes related to Teplizumab’s effects on pancreatic β-cells are identified, and patterns or discrepancies within the literature are discussed.

## Pathophysiology of type 1 diabetes mellitus

T1D stems from the autoimmune destruction of insulin-producing pancreatic beta cells [2]. This process involves beta cell autoantigens, macrophages, dendritic cells, B lymphocytes, and T lymphocytes [17]. Beta cell autoantigens are released through cellular turnover or damage and are presented to T helper cells by antigen-presenting cells. Macrophages and dendritic cells are the initial infiltrators of pancreatic islets [2]. Naive CD4 + T cells in circulation can recognize major histocompatibility complexes and beta cell peptides, leading to interleukin (IL)-12 activation from macrophages and dendritic cells [3]. Simultaneously, beta cell antigen-specific CD8 + T cells are activated by IL-2 from activated TH1 CD4 + T cells, transforming into cytotoxic T cells and entering pancreatic islets [17]. The concerted action of activated TH1 CD4 + T cells, CD8 + cytotoxic T cells, and macrophages contributes to beta cell destruction through granzymes, perforin, cytokines, and reactive oxygen molecules [5]. This collaboration results in autoimmune type 1 diabetes [1, 17].

### Role of the immune system in attacking β-cells

To understand the interactions between beta cells and immune cells in type 1 diabetes (T1D), it is crucial to consider when these cells can physically engage or be exposed to secreted factors. Beta cells are uniquely located in the islets of Langerhans in the pancreas, alongside alpha, delta, epsilon, and pancreatic polypeptide cells [15]. Islets, comprising 1–2% of the pancreas, are separated from acinar cells by connective tissue, granting them “immune privilege.” Under normal conditions, this barrier prevents immune cells in the exocrine pancreas from reaching beta cells. Immune cells must be recruited to islets through chemokines, extravasate, accumulate at the periphery, and break down the barrier to interact with endocrine cells [18].

The most compelling evidence supporting the involvement of the immune system in T1D includes findings of lymphocytic infiltration in the islets of T1D cadaveric donors [19–21], the production of islet-specific autoantibodies in T1D individuals, and studies involving identical twins where the one with T1D rejected islet transplants from their non-diabetic twin [19, 22–25]. Analyses of pancreas sections from T1D individuals reveal pronounced immune infiltration within islets, emphasizing the role of CD4 and CD8 T cells in destroying beta cells [19, 26–28]. This starkly contrasts with pancreas sections from Type 2 Diabetes (T2D) individuals, who, despite having elevated systemic inflammatory markers, lack similar T cell infiltration in pancreatic islets [19, 26–28]. The presence of insulin-specific autoantibodies in nearly all those developing T1D before age 5 underscores the significance of insulin-derived peptides in the disease’s pathogenesis [19, 29, 30]. Islet autoantibodies serve as a diagnostic marker distinguishing T1D from T2D, originating from interactions between autoreactive B cells and autoreactive CD4 T cells [19, 31]. The association of specific human leukocyte antigen (HLA) class II alleles (DR4, DQ8, DQ2) with a heightened genetic risk for T1D implies a crucial role for HLA II-restricted CD4 T cells in the development of the disease, influencing B cell responses, antibody production, CD8 T cell activity, and stimulation of islet-resident macrophages [19, 32, 33].

### Mechanisms leading to β-cell dysfunction and insulin deficiency

The diminished insulin secretion observed in T2D may arise from a decline in individual β-cell function, a decrease in β-cell mass (a product of β-cell size and number), or both. Despite ongoing debates about the relative contributions of secretory dysfunction and loss of β-cell mass to impaired insulin secretion in T2D, a consensus view is yet to emerge [33–37]. Challenges in obtaining high-quality islets for functional studies, especially from T2D donors, contribute to this uncertainty. Various factors, such as differences in drug regimens, genetic backgrounds, and environmental influences, can confound the comparison between control and T2D islet function. However, studies controlling for these variables have consistently shown defective glucose-stimulated insulin secretion (GSIS) in islets from T2D donors [37, 38]. Histological studies indicate a decrease in β-cell mass in T2D, but the extent remains unclear due to challenges in detecting insulin in some β-cells. Evidence suggests a progressive loss of β-cell mass during T2D progression, placing an increasing secretory burden on remaining functional β-cells, influenced by complex interactions of environmental, genetic, and epigenetic factors [33, 38–41].

## **Teplizumab: mechanisms of action**

Teplizumab, an immunotherapy for T1D, operates through diverse mechanisms. It induces phenotypic changes in autoreactive CD8 + T cells, depleting effector T cells (Teffs) while preserving regulatory T cells (Tregs) [42]. Insights from humanized mice suggest the drug’s potential to induce gut-tropic regulatory cells [43]. CD8 + T cell exhaustion, correlating with clinical responses in new-onset T1D, is also associated with teplizumab [13, 44]. Despite these findings, the precise in vivo action targeting peptide-MHC complexes must still be completed [45].

### Targeting specific immune pathways involved in type 1 DM

Teplizumab targets specific immune pathways in T1D by altering the phenotype of autoreactive CD8 + T cells. It favours effector T cell depletion while preserving regulatory T cells [42]. The drug induces gut-tropic regulatory cells, potentially influencing immune responses in the gastrointestinal tract [43]. Trials reveal teplizumab’s positive outcomes, justifying its use, especially in individuals with specific HLA genotypes and serological markers [46]. The drug’s differentiation from otelixizumab, another anti-CD3 antibody, lies in its specificity to the ε-subunit of CD3 [47]. However, the exact basis for drug response and the true mechanism of action remains unresolved [48].

In T1D, the involvement of type I and type II interferon (IFN)-inducible genes suggests the potential role of IFN pathways in the disease’s pathogenesis [49, 50]. The type I IFN pathway in innate immunity is implicated in T1D immunopathogenesis [51]. Discussions on teplizumab’s timing in T1D considered chronic use and its impact on presymptomatic individuals [44].

### Modulation of immune responses and preservation of β-cell function

Teplizumab exhibits promise in preserving β-cell function in recent-onset T1D by altering autoreactive CD8 + T cell phenotypes and depleting effector T cells while preserving regulatory T cells. This modulation is crucial for β-cell function, evidenced by improved and stabilized function post-treatment [42, 52, 53]. Teplizumab is linked to preserving residual β-cell function and reducing exogenous insulin use [47, 54, 55]. This preservation may mitigate short- and long-term complications of T1D [56]. The induction of gut-tropic regulatory cells by teplizumab contributes to its mechanism of action in preserving β-cell function [43]. Notably, the preservation of β-cell function extends beyond the initial treatment period, with improved insulin production persisting for up to five years [53].

## Clinical evidence and studies

### Analysis of trial outcomes related to β-cell preservation and disease progression

In a one-year outcome from a randomized placebo-controlled trial exploring teplizumab’s efficacy in treating T1D, promising results emerged. See Table 1. This trial indicated that teplizumab not only prevents the decline in beta-cell function, as measured by C-peptide but also facilitates glycemic control with reduced insulin doses, especially when administered soon after the diagnosis of T1D in children [47]. Building upon this evidence, a study led by Long and colleagues [57], involving 15 participants, demonstrated that teplizumab treatment resulted in a transient decrease in CD3-positive cells in peripheral blood. The primary clinical outcome focused on preserving C-peptide levels in the plasma, extending the impact to two years after the study initiation.

**Table 1:** Characteristics of included studies

Author & Year	Study Design	Sample Size	Efficacy	Safety profile	Other outcomes
Tooley et al. 2016	2 randomized clinical studies: AbATE trial and Delay trial	125 + 53 = 183 Study size from 2 Kevan Herold study	Clinical responders showed a significant reduction in circulating CD4 + effector memory T cells. Afterward, there was an increase in the frequency and absolute number of CD8CM T cells. BY 2 months: In AbATE trial: (responder: 14.6 ± 2.16% vs. nonresponder: 10.6 ± 1.5%, *p* = 0.13) In Delay trial: (10.0 ± 1.41 vs. 6.25 ± 1.23%, *p* = 0.046). In vitro, teplizumab expanded CD8CM T cells by proliferation and conversion of non-CM T cells.	Nil	-Nanostring analysis of gene expression of CD8CM T cells from responders and non responders versus placebo-treated control subjects identified decreases in expression of genes associated with immune activation and increases in expression of genes associated with T-cell differentiation and regulation. - CD8CM T cells with decreased activation and regulatory gene expression are associated with clinical responses to teplizumab in patients with T1D
Nicole Sherry et al. 2011	2-year trial (protégé study)	763	Teplizumab had a treatment effect on C-peptide and insulin use while maintaining glycaemic control, particularly in selected, prespecified subgroups 54.39%(415), and exemplifies the risks of developing a new outcome without previous validation	77% (318/415) of patients treated with teplizumab (150/207 [72%] in the 14-day full-dose group, 78/102 [76%] in the 14-day low dose group, and 90/106 [85%] in the 6-day full-dose group) and 13% (13/98) of those receiving placebo developed anti-drug antibodies.	- Across the four study groups, similar proportions of patients had adverse events (414/417 [99%] in the teplizumab groups vs. 98/99 [99%] in the placebo group) and serious adverse events (42/417 [10%] vs. 9/99 [9%])
Alice Long et al. 2017	AbATE clinical trial	15	Treatment with teplizumab leads to a transient reduction of CD3-positive cells from peripheral blood, its more durable effects correspond to an agonist activity that induces markers reminiscent of hyporesponsive cells	Nil	- The primary clinical outcome was the preservation of plasma C-peptide two years after initiation of the study measured as a 4-h MMTT-stimulated C-peptide AUC. - Teplizumab increases proportions of central memory cells and cells displaying an exhausted phenotype in circulating CD8 T cell - Teplizumab modulates CD127 expression in circulating CD8 T cell Subsets. - Teplizumab increases proportions of effector memory and PD-1 + cells in circulating CD4 T cells -The observed phenotypic changes across cell types were associated with favorable responses to treatment in the subgroup of study participants who did not develop anti-drug antibodies after the first course of therapy
Jasmin Lebastchi et al. 2013	Clinical trial	93	Higher rates of b-cell death in patients with recent-onset T1D 46.23%(43) versus nondiabetic control subjects 13.98% (13) and have shown that teplizumab treatment is associated with reduced level of b-cell death.	Nil	The levels of unmethylated INS DNA are elevated in subjects with new-onset T1D compared with nondiabetic control subjects and suggest active b-cell destruction at the time of onset of disease.
Kevan C Herold et al. 2013	randomized, open-labeled, controlled trial.	125	− 2 courses of treatment with FcR nonbinding anti-CD3 mAb (teplizumab) reduced the loss of C-peptide 2 years after the first treatment in patients with new-onset T1D. (52)(*p* = 0.002) - teplizumab treatment preserves insulin production and reduces the use of exogenous insulin in some patients with new-onset T1D	Nil	- There was a significant reduction in the titer of anti-ZnT8 antibodies b ut not the other biochemical autoantibodies in the drug-treated group at the end of year 1 (*p* < 0.05) and the differences at 2 years were not statistically significant - There was a total of 11 serious adverse events (SAEs) in 10 drug-treated subjects and 2 SAEs in 1 control subject - clinical responders had lower HbA1c levels while the non-responders does not(received same amount of drugs).
Kevan C. Herold et al. 2009	randomized, open labeled phase IIb trial	10	Study supports previous investigations showing that treatment of patients with new onset T1DM with a brief course of teplizumab results in a reduced rate of decline in insulin production and a decrease in insulin requirements for more than 2 years after diagnosis. A higher dose of drug, approximately 40% higher than the dose used previously, resulted in increased AEs	Nil	- Teplizumab caused transient reduction in circulating T cells, but the recovered cells were not new thymic emigrants because T cell receptor excision circles were not increased. - increased adverse events due to increase dose - There was a trend for reduced loss of C-peptide over 2 years with drug treatment (*p* = 0.1), and insulin use was lower (pb0.001) - Anti-CD3 mAbs may prolong β cell function up to 2 years in patients with new onset Type 1 diabetes (T1DM).
Ana Luisa Perdigoto et al. 2018	Autoimmunity-Blocking Antibody for Tolerance (AbATE) trial	77	C-peptide and T cell markers of response remain in individuals with type 1 diabetes, who were treated with teplizumab, for more than 7 years after diagnosis.	Major adverse events were captured at the time of the follow-up visit	- Drug-treated responders showed a significantly increased frequency of programmed cell death protein 1-positive central memory and anergic CD8 + T cells at follow-up - the drug-treated responders demonstrated a significantly greater C-peptide response than the control group and drug-treated non-responders - Insulin use and HbA1c levels were significantly lower in the drug-treated responders than in the drug-treated non-responders or control participants at visits during the first 2 years but not at the follow-up visit - There was a modest inverse relationship between the C-peptide AUC and daily insulin use - The titres of anti-ZnT8 (*p* < 0.01) and IA-2 antibodies (*p* < 0.001) declined but MIAA (*p* < 0.001) increased over time. The titres of anti-GAD65 antibody did not change significantly.
Kevan C. Herold et al. 2019	phase 2, randomized, placebo-controlled, double-blind trial of teplizumab	76	2-week course of treatment with teplizumab delayed the diagnosis of clinical type 1 diabetes in high-risk participant. -a 2-week course of treatment with teplizumab delayed the diagnosis of clinical type 1 diabetes in high-risk participant; 19 (43%) of the 44 participants who received teplizumab and 23 (72%) of the 32 participants who received placebo had type 1 diabetes diagnosed.	- Teplizumab treatment was associated with adverse events but we’re all later resolve except in one person(Lymphopenia) -Teplizumab delayed progression to clinical type 1 diabetes in high-risk participants.	- There were expected adverse events of rash and transient lymphopenia. - The hazard ratio for the diagnosis of type 1 diabetes (teplizumab vs. placebo) was 0.41 (95% confidence interval, 0.22 to 0.78; *P* = 0.006 - The annualized rates of diagnosis of diabetes were 14.9% per year in the teplizumab group and 35.9% per year in the placebo group.
K. C. Herold et al. 2013	a randomised placebo-controlled trial	58	-The teplizumab group lost significantly less C-peptide at 12 months as a percentage of the C-peptide at baseline (18% [95% CI 7.43, 28.5] vs. 39.0% [95% CI 27.8%, 50.2%], p00.006). At 12 months, five of the 58 participants did not have detectable C-peptide -Teplizumab treatment had no significant effect on the HbA1c levels or on change in the HbA1c levels over the 12-month study period	All adverse events were resolved	- significant increase in CD8CM T cells at month 2 in teplizumab clinical responders compared with teplizumab non-responders (p00.018) -There was a significant difference in the responses of younger (age < 15 years) and older participants (≥ 15 years) (p00.047). When corrected for baseline C-peptide and HbA1c levels, teplizumab-treated younger participants had C-peptide responses that were 31.7% higher than placebo (p00.02) whereas the teplizumab-treated older participants showed no difference (p00.56)

Furthermore, insights from a trial conducted by Lebastchi and colleagues [58], encompassing 93 participants, underscored that teplizumab treatment correlated with a reduction in beta-cell death in the pancreas. Shifting the focus to a randomized open-labeled controlled trial by Herold and colleagues [59] with 125 subjects, the data suggested that two courses of teplizumab substantially curbed the loss of C-peptide two years after the initial treatment in patients with new-onset T1D. The overarching conclusion pointed towards teplizumab preserving insulin production and minimizing the need for exogenous insulin in these patients.

This conclusion was corroborated by another trial by Herold and colleagues in 2009 [53], involving 10 subjects in a randomized open-labeled phase IIb trial. The study reinforced previous reports, showcasing that a brief course of teplizumab led to a sustained decline in insulin production and requirements for over two years post-diagnosis. However, a cautionary note emerged regarding an increased drug dose, approximately 40% above the normal dose, which resulted in adverse events. In a distinct phase, 2 randomized double-blinded study featuring a 2-week course of teplizumab treatment, it was reported that teplizumab delayed the onset of Type 1 Diabetes among high-risk individuals compared to the placebo group [60]. Additionally, the study indicated a delay in the progression to clinical diabetes in high-risk participants.

A subsequent randomized placebo-controlled trial involving 58 participants demonstrated that the teplizumab group experienced significantly less loss of C-peptide at 12 months. However, it was noteworthy that five participants in this group had no detectable C-peptide level. The study also reported that while teplizumab treatment did not significantly affect HbA1c levels, its effectiveness varied based on age, being more pronounced among younger participants compared to the placebo group. Conversely, teplizumab-treated older participants showed no significant difference [61].

In a pivotal phase 3, the randomized, placebo-controlled trial led by Ramos et al. in 2023 [62], which assessed beta-cell preservation, clinical endpoints, and safety in children and adolescents, two 12-day courses of teplizumab showed significant benefits in preserving beta-cell function. However, no significant differences between groups were observed concerning secondary endpoints. In a similar study by Sims et al., 2021 [52], teplizumab changed the course of the disease. It increased the function of beta cells, which was observed as qualitative and quantitative improvements in insulin secretion. It increased OGTT and reversed the decline of C-peptide levels during the first 6 months of treatment. The cumulative evidence paints a promising picture for teplizumab, suggesting its potential to preserve beta-cell function and effectively manage glycemic control, especially when administered early in T1D.

### Discussion of efficacy, safety, and long-term effects observed in trials

Adverse effects associated with teplizumab administration encompassed headache, gastrointestinal symptoms, lymphopenia, leukopenia, decreased blood bicarbonates, rash, and mild cytokine release syndrome [63]. Notably, infection rates were comparable between teplizumab-treated and control patients, and the emergence of the new Epstein-Barr virus was reported in the former. Additionally, hypersensitivity reactions such as anaphylaxis, angioedema, urticaria, and peripheral oedema were observed in teplizumab-treated patients [64]. While the long-term effects of teplizumab are still under scrutiny, multiple trials, including an integrated efficacy analysis, demonstrated a significant preservation of C-peptide levels in patients treated with teplizumab compared to controls one to two years after treatment. This preservation was coupled with a higher C-peptide level, indicative of improved exogenous insulin production, and supported by a decreased use of exogenous insulin [65].

A pivotal study by Sims and colleagues [52] highlighted that teplizumab influenced the biological course of Type 1 Diabetes. The treatment was associated with quantitative and qualitative improvements in insulin secretion, and these changes were linked with the modulation of the function and frequency of memory CD9 + T cells. The early efficacy of teplizumab followed by beta cell function stabilization suggests the potential benefit of repeat treatments with other regimens [55, 63] or additional drugs directly acting on beta cells to extend the delay or prevent the diagnosis of T1D.

### Examination of Teplizumab’s influence on preserving β-cell mass function

Pancreatic β-cell dysfunction is a pivotal factor in the initiation and progression of T1D, involving altered β-cell function and reduced β-cell mass. These dysfunctions contribute to characteristic insulin release defects, leading to a gradual elevation in glucose levels and a decline in glycemic control over time. Certain therapies show promise in delaying diabetes onset and slowing glycemic control deterioration, with evidence pointing to the preservation of β-cell function as a contributing factor [66].

Teplizumab, a subject of interest for over two decades, is believed to influence the progression of Type 1 Diabetes from stage 2 to stage 3 by preserving β-cell function. This influence has been consistently demonstrated in various clinical trials, where C-peptide levels, indicative of β-cell function, were maintained [67]. In a recent phase 3 randomized-controlled trial involving children and adolescents, individuals treated with Teplizumab exhibited significantly higher stimulated C-peptide levels compared to the placebo group at week 78. This indicates a notable preservation of β-cell function, suggesting that Teplizumab positively impacts insulin production in these patients [68].

Moreover, an integrated analysis of five trials in patients with stage 3 T1D further supports the efficacy of Teplizumab. The analysis revealed that one or two courses of Teplizumab led to a significant improvement in stimulated C-peptide levels, a reduction in exogenous insulin use, and the preservation of β-cell function compared to the placebo at 1 and 2 years of follow-up. This consistent preservation of β-cell function, as indicated by C-peptide levels, underscores the potential of Teplizumab in influencing the course of T1D [69].

The retention of insulin production facilitated by Teplizumab, even beyond the new-onset period, has been associated with a more favourable prognosis. This includes reduced rates of complications such as retinopathy, nephropathy, neuropathy, and hypoglycemia. The suggestion is that Teplizumab treatment may offer several clinical benefits by arresting the decline in insulin production, providing a promising avenue for improving outcomes in individuals with T1D [63].

## Analysis of markers indicating β-cell health post-teplizumab therapy

Experimental evidence highlights that individuals responding to teplizumab therapy for diabetes exhibit a reduced decline in C-peptide and sustained immunological responses after diagnosis [70]. The Autoimmunity-Blocking Antibody for Tolerance (AbATE) trial, in a 7-year follow-up study, demonstrated that drug-treated responders exhibited a significantly greater C-peptide response than the control and drug-treated non-responder groups. Despite a decline, C-peptide responses remained consistently higher in responders than non-responders and controls, suggesting a favourable impact of Teplizumab on preserving β-cell function [70].

In the TrialNet Anti-CD3 Prevention (TN10) trial, teplizumab significantly delayed the development of clinical T1D, with a remarkable 50% of treated individuals remaining free of diabetes compared to only 22% in the placebo group [71]. Collectively, evidence from the AbATE and TN10 trials underscores the promising outcomes of Teplizumab therapy in preserving β-cell function and delaying the onset of clinical T1D. The nuanced effects observed in the AbATE trial prompt further investigation into the diverse impact of Teplizumab on diabetes progression [70, 71].

### Correlation between β-cell preservation and glycemic control

Despite advancements in diabetes management, achieving optimal glycemic control in T1D remains challenging. Individuals maintaining endogenous beta-cell function for extended periods post-diagnosis experience lower HbA1c levels, reduced hypoglycemia, and a decreased incidence of long-term complications [72].

Residual pancreatic beta-cell function, indicated by stimulated C-peptide secretion, correlates with a diminished risk of complications. The persistence of endogenous insulin secretion, even in small amounts, is associated with improved metabolic control, evident by a 1% lower HbA1c, reduced ketosis risk, and fewer hypoglycemic instances. At diagnosis, the potential for spontaneous insulin-free remission is heightened with glucagon-stimulated C-peptide levels > 400 pmol/L [71]. A study by Theodorus et al. demonstrated a positive correlation between β-cell preservation, measured by extending PET, and glycemic control in T1D. Higher β-cell mass was associated with better glycemic stability, emphasizing the importance of preserving β-cell mass for improved glycemic outcomes [72].

Good initial metabolic control consistently preserves beta-cell function, emphasizing the urgency of intensive management in early Type 1 Diabetes. New immunotherapies, like Teplizumab, aimed at preserving residual beta-cell function, are particularly significant when initiated early. Early treatments may preserve function long enough to introduce more effective second-generation treatments, offering substantial long-term benefits, especially for those facing challenges in managing glycemic control [72].

## Clinical implications and future directions

The recent discovery of Teplizumab’s beneficial effects on β-cell activity in the pancreas paves the way for new theoretical avenues and necessitates a comprehensive investigation into its real-world implications for altering the treatment plan of T1D [52]. A thorough assessment of the theoretical and practical aspects of Teplizumab is crucial. Questions about the sustainability of its effects over time, its impact on glycemic control, and its potential as a stand-alone treatment or in combination with other therapies must be addressed [62]. Despite Teplizumab’s promising potential, its real-world application may encounter obstacles and restrictions. Considerations such as potential side effects [67], patient selection criteria [70], and logistical challenges should be considered when contemplating the integration of Teplizumab into the therapy regimen for T1D. Ignoring these obstacles could impede the seamless adoption of Teplizumab in T1D treatment plans.

Expanding beyond traditional monotherapies is essential in the future, given the dynamic nature of diabetes research. Investigating combination therapies involving Teplizumab and other medications emerges as a plausible path to harness synergistic effects. The development of personalized treatment strategies tailored to the unique characteristics of each patient is imperative to maximize Teplizumab’s benefits while minimizing potential side effects.

## Strengths and limitations of review

The review provides a comprehensive and up-to-date analysis of Teplizumab’s impact on β-cell preservation and its potential role in T1D treatment. It synthesizes evidence from various clinical trials, offering a nuanced perspective on Teplizumab’s efficacy. The review is contingent on the available English-language literature up to its last update and may not capture recent developments in non-English studies. While the review addresses theoretical and practical aspects, further research may be needed to validate the findings in diverse patient populations.

## Conclusion

Teplizumab is more effective in preventing beta-cell function loss as evaluated by C-peptide and providing glycemic control at lower insulin doses if administered to patients before or after the diagnosis of T1D. Teplizumab, an FDA-approved immunomodulatory medicine, takes a big step toward delaying the onset of T1D by regulating the immune system by targeting the CD3 antigen while preserving residual beta-cell function.

Clinical trials have shown that Teplizumab effectively maintains C-peptide levels, lowers insulin dependence, and delays T1DM progression. Although these immunotherapies have improved C-peptide responses, permanent or even long-term maintenance of C-peptide has yet to be accomplished. In addition, challenges such as potential adverse effects, patient selection criteria, and logistical issues must be addressed for real-world deployment. As a result, emerging technologies for viewing and quantifying beta mass and killing may be beneficial in determining beta cell changes that cause the disease and those that occur after immune therapy. Future research will necessitate the examination of human materials and trials that combine drugs capable of modulating immune responses with those that directly interfere with beta cell destruction while augmenting their responses.

## Data Availability

Data sharing is not applicable to this article as no datasets were generated or analyzed during the current study.

## Abbreviations

T1D: Type 1 Diabetes Mellitus
β-cells: Beta Cells
FDA: Food and Drug Administration
CD3: Cluster of Differentiation 3
TCR: T-Cell Receptor
mAb: Monoclonal Antibody
IL: Interleukin
GAD65: Glutamic Acid Decarboxylase Isoform 65
IAA: Insulin Antibodies
IA-2: Insulinoma Antigen 2
ZnT8: Zinc Transporter Isoform 8
MDI: Multiple Daily Insulin Injections
CGM: Continuous Glucose Monitoring
IFN: Interferon
HLA: Human Leukocyte Antigen
Tregs: Regulatory T Cells
AbATE: Autoimmunity-Blocking Antibody for Tolerance
TN10: TrialNet Anti-CD3 Prevention
PET: Positron Emission Tomography
